# Newcastle disease virus-attenuated vaccine LaSota played a key role in the pathogenicity of contaminated exogenous virus

**DOI:** 10.1186/s13567-018-0577-z

**Published:** 2018-08-06

**Authors:** Qi Su, Yang Li, Yawen Zhang, Zhihui Zhang, Fanfeng Meng, Zhizhong Cui, Shuang Chang, Peng Zhao

**Affiliations:** 1 College of Veterinary Medicine, Shandong Agricultural University, 61 Daizong Street, Tai’an, 271018 Shandong China; 2 Shandong Provincial Key Laboratory of Animal Biotechnology and Disease Control and Prevention, 61 Daizong Street, Tai’an, 271018 Shandong China; 3 Shandong Provincial Engineering Technology Research Center of Animal Disease Control and Prevention, 61 Daizong Street, Tai’an, 271018 Shandong China; 4 China Animal Health and Epidemiology Center, 369 Nanjing Street, Qingdao, 266000 Shandong China

## Abstract

Newcastle disease virus (NDV)-attenuated vaccine has been widely used since the 1950s and made great progress in preventing and controlling Newcastle disease. However, many reports mention exogenous virus contamination in attenuated vaccines, while co-contamination with fowl adenovirus (FAdV) and chicken infectious anaemia virus (CIAV) in the NDV-attenuated vaccine also emerged in China recently, which proved to be an important reason for the outbreaks of inclusion body hepatitis–hydropericardium syndrome in some flocks. It is amazing that exogenous virus contamination at extremely low doses still infected chickens and induced severe disease; thus, we speculated that there must be some interaction between the NDV-attenuated vaccine and the contaminated exogenous viruses within. Accordingly, simulation experiments were launched using FAdV and CIAV isolated from the abovementioned vaccine. The results showed that the pathogenicity of FAdV and CIAV co-infection through the contaminated vaccine was significantly higher than that of direct oral infection, while the synergistic reaction of these viruses and LaSota prompted their multiplication in vivo and disturbed the production of antibodies against each other. This study showed the interactions of FAdV, CIAV and LaSota after using contaminated NDV-attenuated vaccine, helping us to understand how the contaminated exogenous viruses cause infection and induce severe disease at a relatively low dose through the oral route.

## Introduction

The World Organization for Animal Health has defined Newcastle disease (ND) as the infection of poultry with virulent strains of Newcastle disease virus (NDV). Lesions affecting the neurological, gastrointestinal, respiratory, and reproductive systems are most often observed [1–3]. The control of ND must include strict biosecurity that prevents virulent NDV from coming in contact with poultry, as well as proper administration of efficacious vaccines. When administered correctly to healthy birds, ND vaccines formulated with NDV of low virulence or viral-vectored vaccines that express the NDV fusion protein are able to prevent clinical disease and mortality in chickens upon infection with virulent NDV. Live vaccines have been widely used since the 1950s and have made great progress in preventing and controlling ND [4]. However, many reports mention exogenous virus contamination in attenuated vaccines, and we also found co-contamination with fowl adenovirus (FAdV) and chicken infectious anaemia virus (CIAV) in the NDV-attenuated vaccine, which proved to be an important reason for an outbreak of inclusion body hepatitis–hydropericardium syndrome (IBH–HPS) in some flocks [5].

With the advancement of poultry breeding conditions, strict biosecurity in most farms has been sufficient to resist many infectious diseases. However, exogenous virus contamination in attenuated vaccines emerged in an endless stream [6–18], which even broke through multiple barriers and caused the outbreak of some severe diseases on some farms [5, 19]. Potential risks induced by exogenous virus contamination in attenuated or live vaccines are worthy of attention, but the role that the vaccine plays in such diseases equally needs further research and assessment. Although some reports mentioned interactions between the NDV vaccine and CIAV [20–22], the latter did not cause infection through the use of contaminated vaccines. The contamination dose in attenuated vaccines is extremely low, and oral infection is also not a good way for FAdV and CIAV to invade the chicken. We speculated that there must be some interactions between the NDV-attenuated vaccine and the contaminated exogenous viruses within, which might be critical factors for FAdV and CIAV infection of chickens causing severe disease. Thus, systemic animal experiments were performed to show differences between FAdV and CIAV infection via contaminated vaccine, as well as direct infection through the mouth.

## Materials and methods

### Virus background

In the second half of 2015, the highest mortality from severe IBH–HPS appeared on a large-scale poultry farm, while detection results showed co-contamination of FAdV and CIAV for the same NDV-attenuated vaccine. Then, the CIAV vaccine isolate SDAUC-Vac (GenBank Accession Number MF614011) and FAdV vaccine isolate SDAUF-Vac (GenBank Accession Number MF614012) were isolated and purified from this vaccine in our previous study [5]. Subsequently, both the FAdV isolate and CIAV isolate were inoculated into 10-day-old embryonated eggs through the yolk sac to obtain larger stocks. The 50% embryo infectious dose (EID_50_) of these stocks was calculated by the method of Reed-Muench [23], and the stocks were stored at −80 °C for further analysis.

### Animal experimental design

One-day-old SPF chickens (SPAFAS poultry company, Jinan, China) were wing banded and randomly divided into six groups. Each group had 15 chickens, which were separately bred in shielded cages with positive filtered air. Using SDAUC-Vac and SDAUF-Vac, different contaminated vaccines or virus diluents were artificially prepared as trial samples to vaccinate chicks orally at 7 days of age. Specifically, the NDV-attenuated vaccine (all negative in the abovementioned pathogen test) was diluted with phosphate buffered saline (PBS) according to the instructions, and the final volume of each plume was approximately 200 µL. Afterward, FAdV or CIAV isolates were diluted with PBS to 50 EID_50_/µL and 10 EID_50_/µL and then added to each plume of diluted vaccine or an equal amount of normal saline. Specific groups and treatments are shown in Table 1.

**Table 1:** Animal experimental design

Groups	Vaccination methods	Vaccine	Vaccine dosage	Contaminated virus	Viral dosage	Chicken amount	Age/days
LaSota	Orally	NDV	A plume	No virus		15	7
PBS	Orally	PBS	A plume	No virus		15	7
LaSota/FAdV/CIAV/HD	Orally	NDV	A plume	FAdV + CIAV	50 EID_50_ + 50 EID_50_	15	7
LaSota/FAdV/CIAV/LD	Orally	NDV	A plume	FAdV + CIAV	10 EID_50_ + 10 EID_50_	15	7
PBS/FAdV/CIAV/HD	Orally	PBS	A plume	FAdV + CIAV	50 EID_50_ + 50 EID_50_	15	7
PBS/FAdV/CIAV/LD	Orally	PBS	A plume	FAdV + CIAV	10 EID_50_ + 10 EID_50_	15	7

H represents high dose, such as 50 EID_50_; L represents low dose, such as 10 EID_50_.

### Measurement of body weight and immune organ indices

To compare the effects of viral infection on growth retardation and immunosuppression, the growth and death of the chicks were measured daily until 6 weeks of age, as the disease period of IBH–HPS is mainly 3–6 weeks in most poultry flocks. Individual body weights (BWs) were measured at weeks 1, 2, 3, 4, 5, and 6 in the six groups of the experiment. At 3 weeks of age, three chicks from each group were randomly chosen and euthanized with intravenous pentobarbital sodium (New Asia pharmaceutical, Shanghai, China), and tissues were collected, including the heart, liver, thymus, spleen, and bursa of Fabricius, and preserved at −80 °C. At 6 weeks of age, samples of thymus, spleen, and bursa of Fabricius were excised from all chicks and weighed to establish immune organ indices. After weighing the organs, the immune organ indices of the thymus, spleen, and bursa of Fabricius were calculated as organ weight (wet weight, mg)/BW (g) × 100%.

### Quantification of viral load

At 3 weeks of age, three chicks in each group were randomly chosen and euthanized, and tissues were collected, including the lung, weasand, heart, liver, thymus, spleen, and bursa of Fabricius, for RNA extraction (Omega Bio-Tek, Norcross, GA, USA) to determine the LaSota viral load in different organs according to previously published methods [24]. Meanwhile, genomic DNA was also extracted from these organs using a DNA extraction kit (Omega Bio-Tek) for the quantification of FAdV and CIAV viral load using published qPCR methods [25, 26]. Anticoagulated blood was collected from all chicks at 10, 14, 21, 28, and 35 days post-hatching for DNA extraction to determine the viraemia level of FAdV and CIAV. To make the LaSota, FAdV and CIAV copy numbers in the above tissue conform, viral RNA or DNA concentration (log_10_) was normalized per 1 µg of total RNA or DNA. The qPCR reactions were set up on ice, and an ABI PRISMR 7500 Sequence Detection System (Applied Biosystems, USA) was used to amplify and detect the reaction products. The qPCR was performed in duplicate, with each sample present in technical duplicate during each run.

### Antibody responses to NDV, FAdV and CIAV

Sera from all chicks were collected at weeks 2, 3, 4, 5, and 6 of the experiment for haemagglutination inhibition testing to determine the concentrations of NDV antibodies in accordance with routine procedures, while the FAdV and CIAV antibody levels of these samples were also determined using the FAdV Group I antibody test kit (BioChek, Netherlands) and CIAV antibody test kit (Zoetis, USA) according to the instructions.

### Statistical analysis

Statistical analyses were performed using the SPSS statistical software package for Windows version 17.0 (SPSS, Inc., Chicago, Illinois, USA). *P* < 0.05 was considered statistically significant based on Duncan’s multiple-range test.

### Ethics statement

The animal care and use protocol was approved by the Shandong Agricultural University Animal Care and Use Committee (SDAUA-2016-002). All the experimental animals in this study were cared for and maintained throughout the experiments strictly following the ethics and biosecurity guidelines approved by the Institutional Animal Care and Use Committee of Shandong Agricultural University.

## Results

### LaSota is a lethal factor in contaminated vaccine-induced diseases

**Figure 1:**
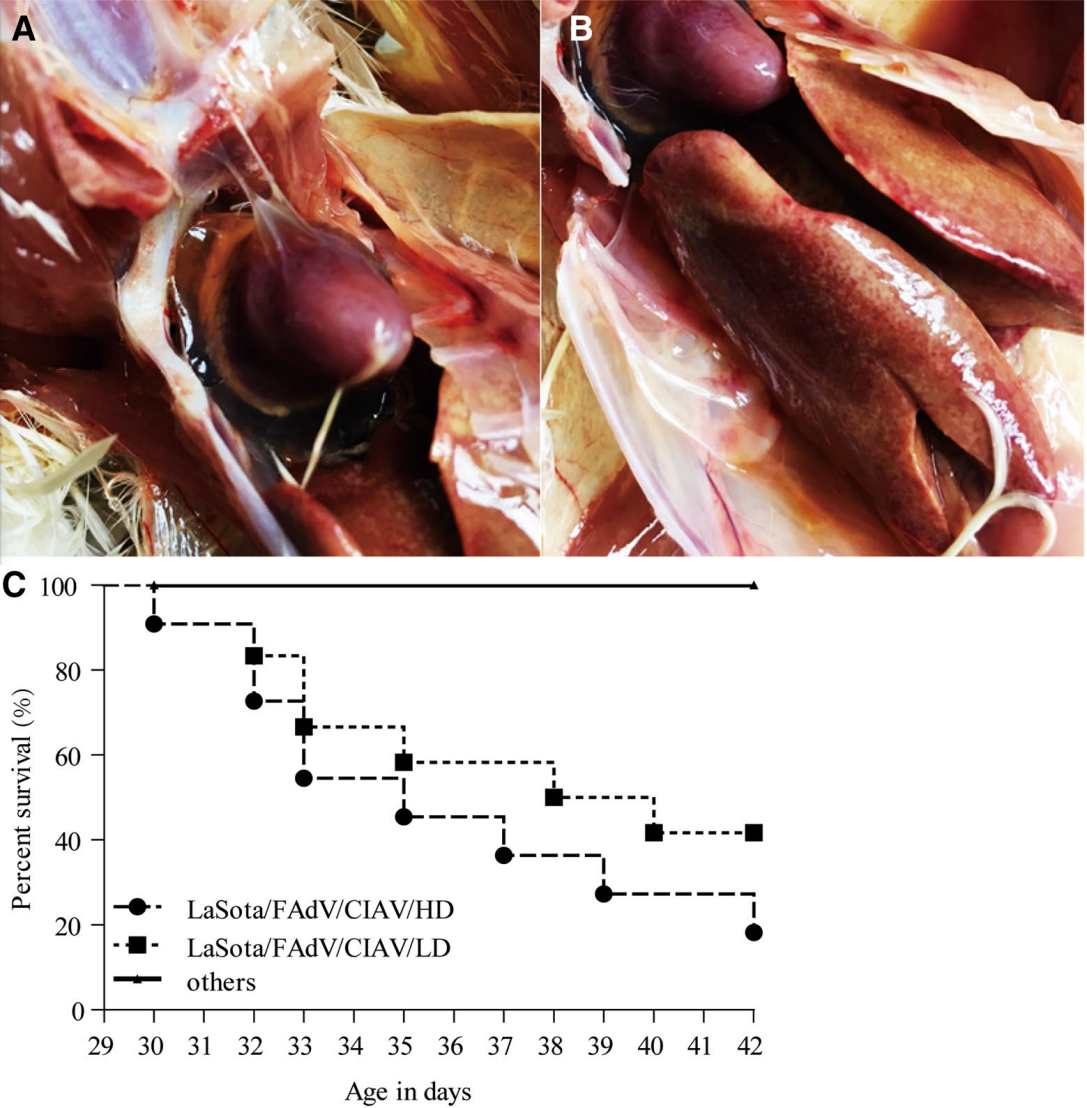
**Clinical symptoms and percent survival curve. A** HPS, the accumulation of clear, straw-coloured fluid in the pericardial sac. **B** IBH, the discoloured swollen liver with pinpoint haemorrhage of dead chicks. **C** Percent survival of each group (these data have been published in a previous study [5]).

To assess the effects of LaSota on contaminated vaccine-induced diseases, we observed and recorded the mortality rates and dissection symptoms of all chickens in these six groups. Specifically, the mortality rates of the LaSota/FAdV/CIAV//HD and LaSota/FAdV/CIAV/LD groups were 75% (9/12, excluding three chicks dissected at 3 weeks of age, 50 EID_50_ per dose) and 58.3% (7/12, 10 EID_50_ per dose), respectively, and death appeared during 30–42 days of age, while no chick died in the other groups (Figure 1C, these data have been published in a previous study [5]). HPS (Figure 1A) appeared in approximately 70% of dead chicks, and typical IBH was shown in all dead chicks (Figure 1B).

### LaSota significantly elevated the growth inhibition caused by FAdV and CIAV

**Figure 2:**
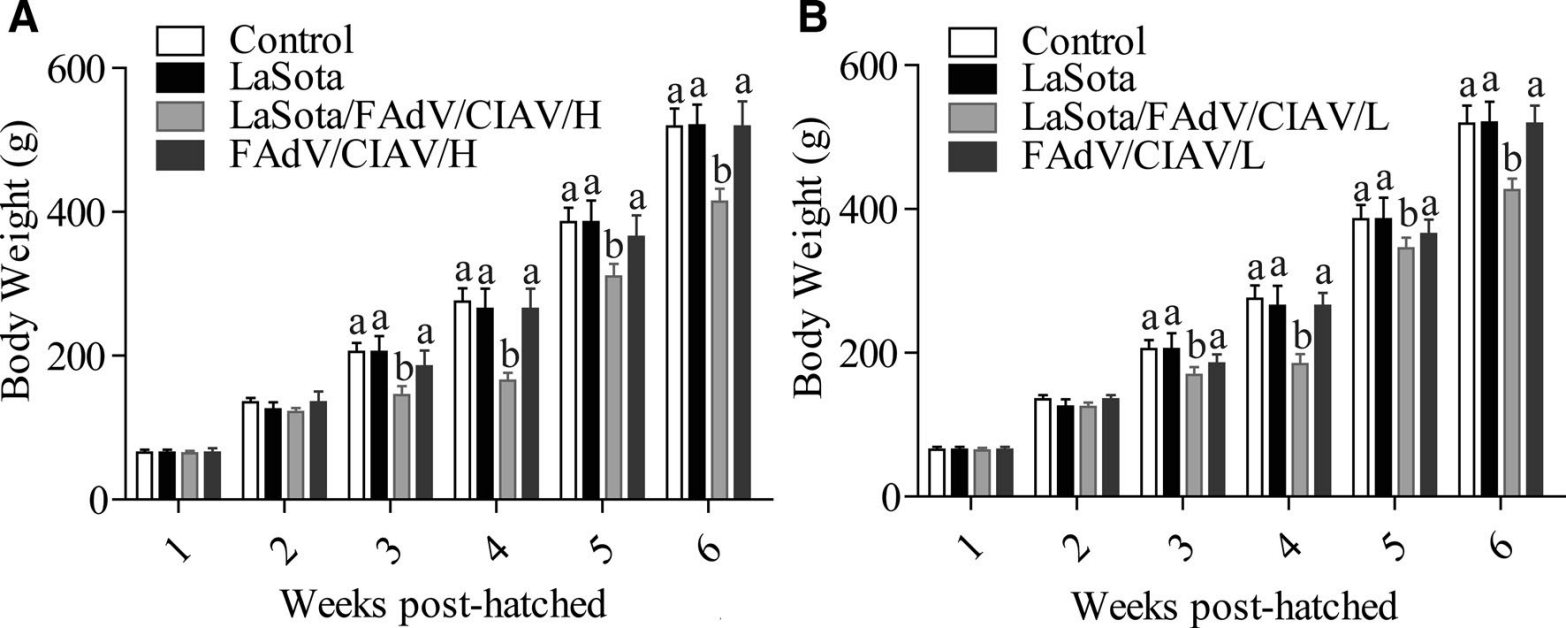
**The body weights of chickens in each group. A** Body weights (mean ± error) of the high-dose experiment. **B** Body weights (mean ± error) of the low dose experiment. The body weight of the chickens in different groups was measured at 1, 2, 3, 4, 5, and 6 weeks post-hatching to evaluate the effect of contaminated vaccine on growth rates. The body weights of different groups at the same time point were analysed by Duncan’s multiple-range test. ^a,b,c,d^The different letters represent significant differences (*P* < 0.05). The same letters indicate that the differences were not significant (*P* > 0.05). Data for the LaSota, LaSota/FAdV/CIAV/HD and LaSota/FAdV/CIAV/LD groups have been published in a previous study [5].

In this study, we compared the BWs of SPF chickens under different treatments to show the effects of different agents on growth rate. In the high dose experiment (Figure 2A), no significant difference was observed between the PBS group and the LaSota group (*P* > 0.05), which demonstrated that normal vaccination has a low impact on the BW growth of SPF chickens. Additionally, the BWs of chickens in the PBS group and the LaSota group were also similar to those in the PBS/FAdV/CIAV/HD group and PBS/FAdV/CIAV/LD group with little difference (*P* > 0.05). However, the BWs of chickens in the LaSota/FAdV/CIAV/HD group were significantly lower than those in the PBS/FAdV/CIAV/HD group starting at 3 weeks of age (*P* < 0.05), which illustrated that the interaction of exogenous viruses and LaSota aggravated the inhibitory action for BW growth of SPF chickens. Meanwhile, the results of the low-dose experiment were consistent with the above results (Figure 2B).

### LaSota elevated the lesions in immune system organs caused by FAdV and CIAV

**Figure 3:**
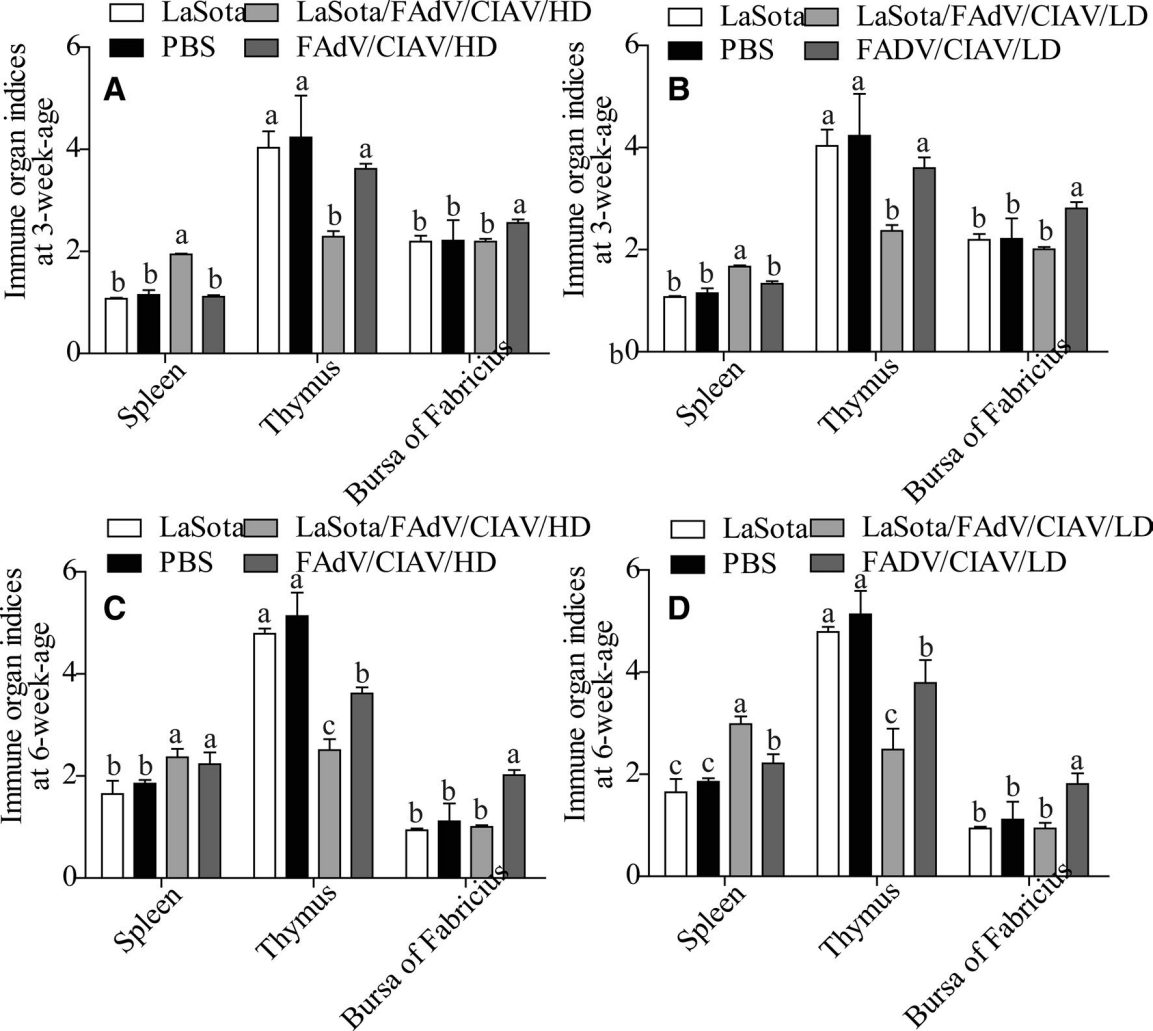
**The results of immune organ indices. A** (High-dose experiment), **B** (low-dose experiment): ratios of organ weight/body weight × 1000 (mean ± error) at 3 weeks of age; organs were harvested from three random chicks. **C** (High-dose experiment), **D** (low-dose experiment): ratios of organ weight/body weight × 1000 (mean ± error) at 6 weeks of age; organs were harvested from all chicks. ^a,b,c,d^The different letters represent significant differences (*P* < 0.05) based on Duncan’s multiple-range test, and the same letters indicate that the differences were not significant (*P* > 0.05). Data for the LaSota, LaSota/FAdV/CIAV/HD and LaSota/FAdV/CIAV/LD groups have been published in a previous study [5].

Both the FAdV and CIAV could affect the development of the immune system. Thus, immune organ indices were employed to show the effects of LaSota on immunosuppression caused by FAdV and CIAV. Both in the high dose experiment (Figure 3A) and low dose experiment (Figure 3B) at 3 weeks of age, chickens in the LaSota group exhibited slight atrophy in the thymus, spleen and bursa of Fabricius compared to chickens from the PBS group (*P* > 0.05), which demonstrated that LaSota could also disturb the development of immune organs mildly in normal vaccination. The immune system organs of other groups showed varying degrees of injury, including splenomegaly and atrophy of the thymus gland, while such injury in the LaSota/FAdV/CIAV/HD group and the LaSota/FAdV/CIAV/LD group was significantly higher than that in the PBS/FAdV/CIAV/HD group and the PBS/FAdV/CIAV/LD group (*P* < 0.05), which showed that LaSota elevated the lesions in immune system organs caused by FAdV and CIAV. On the other hand, compared with the other groups, the bursa of Fabricius in the PBS/FAdV/CIAV/HD group and the PBS/FAdV/CIAV/LD group showed different degrees of swelling (*P* < 0.05). The results at 6 weeks of age were in accordance with the above results (Figures 3C and D).

### LaSota elevated the viral load of FAdV and CIAV in some organs

**Figure 4:**
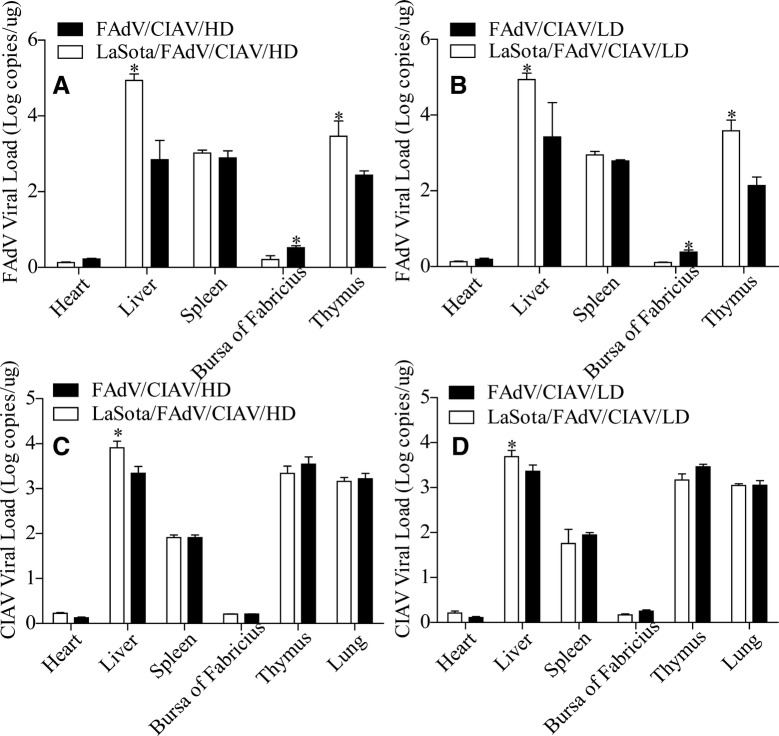
**FAdV and CIAV viral loads in different organs.** Distribution and viral load of FAdV in different organs (**A** high-dose; **B** low-dose), and distribution and viral load of CIAV in different organs (**C** high-dose; **D** low-dose). *Indicates significant difference (*P* < 0.05) between the two experimental groups. Data for the LaSota, LaSota/FAdV/CIAV/HD and LaSota/FAdV/CIAV/LD groups have been published in a previous study [5].

The distributions of FAdV and CIAV in different organs were also determined to show the changes of their tissue tropisms and clinical symptoms under the effects of LaSota. The results showed that compared with the PBS/FAdV/CIAV/HD and PBS/FAdV/CIAV/LD group, the FAdV viral loads of the liver and thymus in the LaSota/FAdV/CIAV/HD and LaSota/FAdV/CIAV/LD groups were obviously elevated (*P* < 0.05, Figures 4A and B), while the CIAV viral loads of the liver in these groups were also elevated (*P* < 0.05, Figures 4C and D), all of which demonstrated that LaSota could elevate the viral load of FAdV and CIAV in some organs.

### LaSota elevated the viraemia levels of FAdV and CIAV

**Figure 5:**
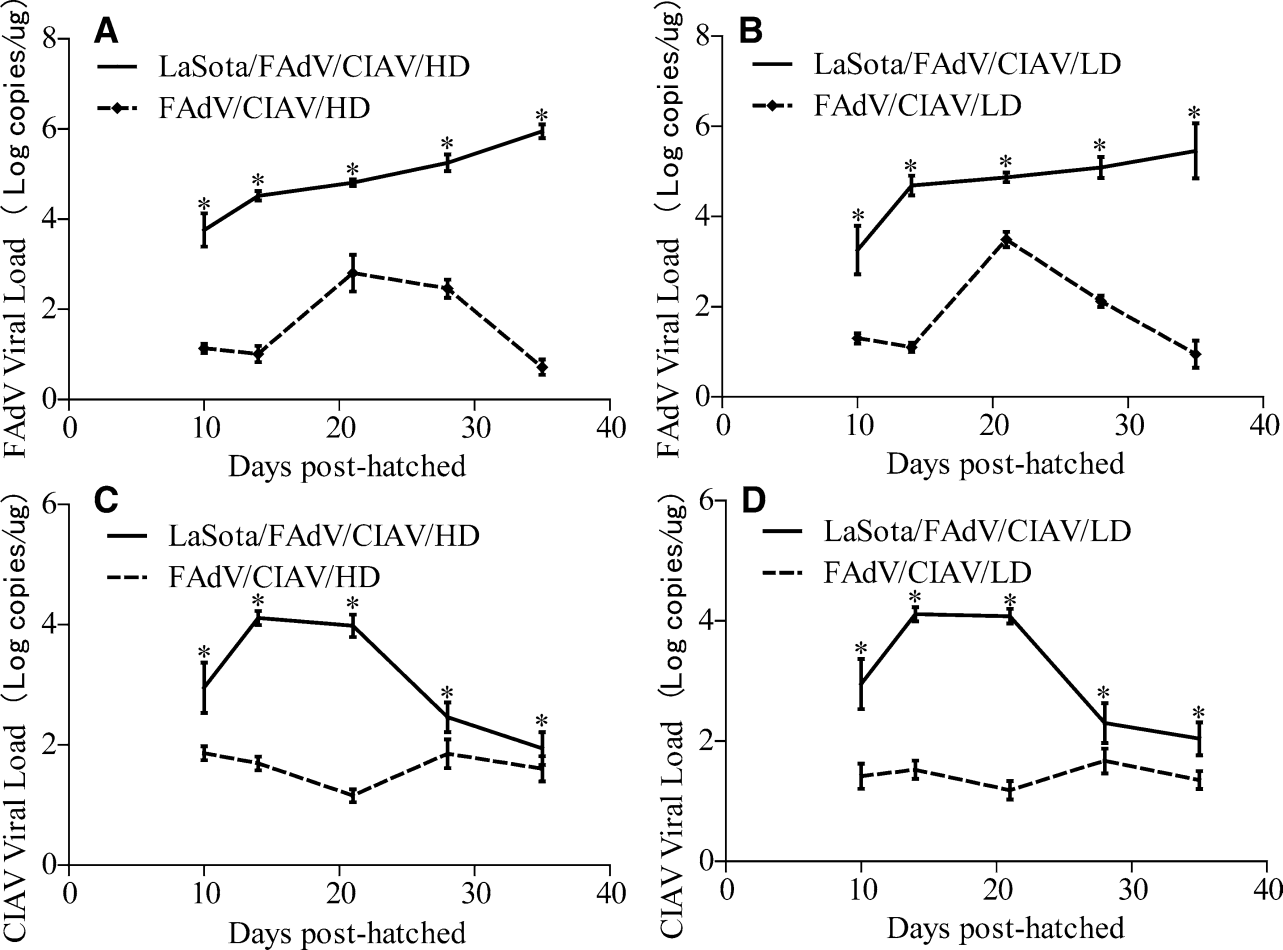
**The viraemia levels of FAdV (A high-dose; B low-dose) and CIAV (C high-dose; D low-dose).** *Indicates significant difference (*P* < 0.05) between the two experimental groups. Data for the LaSota, LaSota/FAdV/CIAV/HD and LaSota/FAdV/CIAV/LD groups have been published in a previous study [5].

To assess the effects of LaSota on FAdV and CIAV and discover why their simultaneous infection caused severe disease and high mortality, we determined the viraemia levels of FAdV and CIAV in different groups. The results showed that the FAdV viraemia levels of the LaSota/FAdV/CIAV/HD and LaSota/FAdV/CIAV/LD groups were significantly higher than those of the PBS/FAdV/CIAV/HD and PBS/FAdV/CIAV/LD groups (*P* < 0.05), respectively. Meanwhile, the viraemia level of the former continued to rise but that of the latter increased first and then decreased over time (Figures 5A and B), which demonstrated that LaSota not only elevated the viraemia level of FAdV but also disturbed the defence system against it. On the other hand, the CIAV viraemia levels of the LaSota/FAdV/CIAV/HD and LaSota/FAdV/CIAV/LD groups were also significantly higher than those of the PBS/FAdV/CIAV/HD and PBS/FAdV/CIAV/LD groups (*P* < 0.05), respectively (Figures 5C and D). More importantly, the FAdV or CIAV viraemia levels of the LaSota/FAdV/CIAV/HD and LaSota/FAdV/CIAV/LD groups were already very high at 3 days post-vaccination, and we speculated that the presence of LaSota may promote the invasion of FAdV and CIAV after vaccination.

### LaSota decreased the antibody responses against FAdV and CIAV

**Figure 6:**
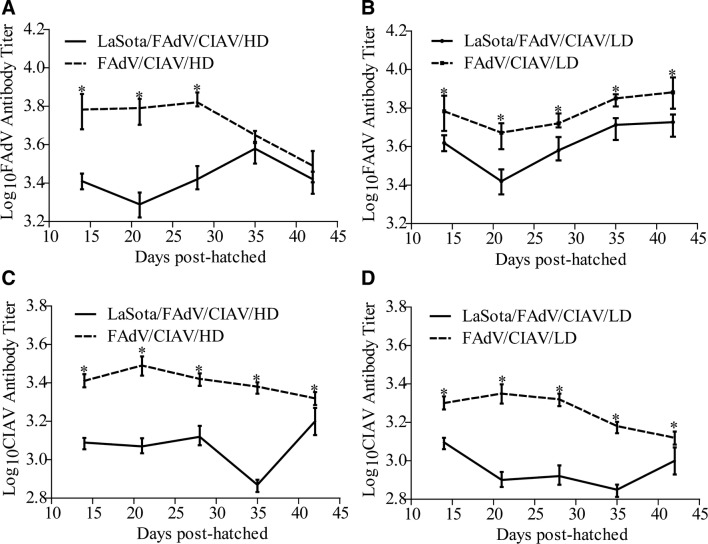
**The antibody levels of FAdV (A high-dose; B low-dose) and CIAV (C high-dose; D low-dose).** *Indicates significant difference (*P* < 0.05) between the two experimental groups.

Sera from all chicks were collected at weeks 2, 3, 4, 5, and 6 of the experiment to determine the concentrations of FAdV and CIAV antibody. The results showed that the FAdV or CIAV antibody levels of the LaSota/FAdV/CIAV/HD or LaSota/FAdV/CIAV/LD groups were significantly higher than those of the corresponding PBS/FAdV/CIAV/H or PBS/FAdV/CIAV/L groups (*P* < 0.05, Figures 6A–D), which demonstrated that LaSota could disturb and decrease the production of FAdV or CIAV antibodies.

### Exogenous viruses activated the pathogenicity and viral load of LaSota

**Figure 7:**
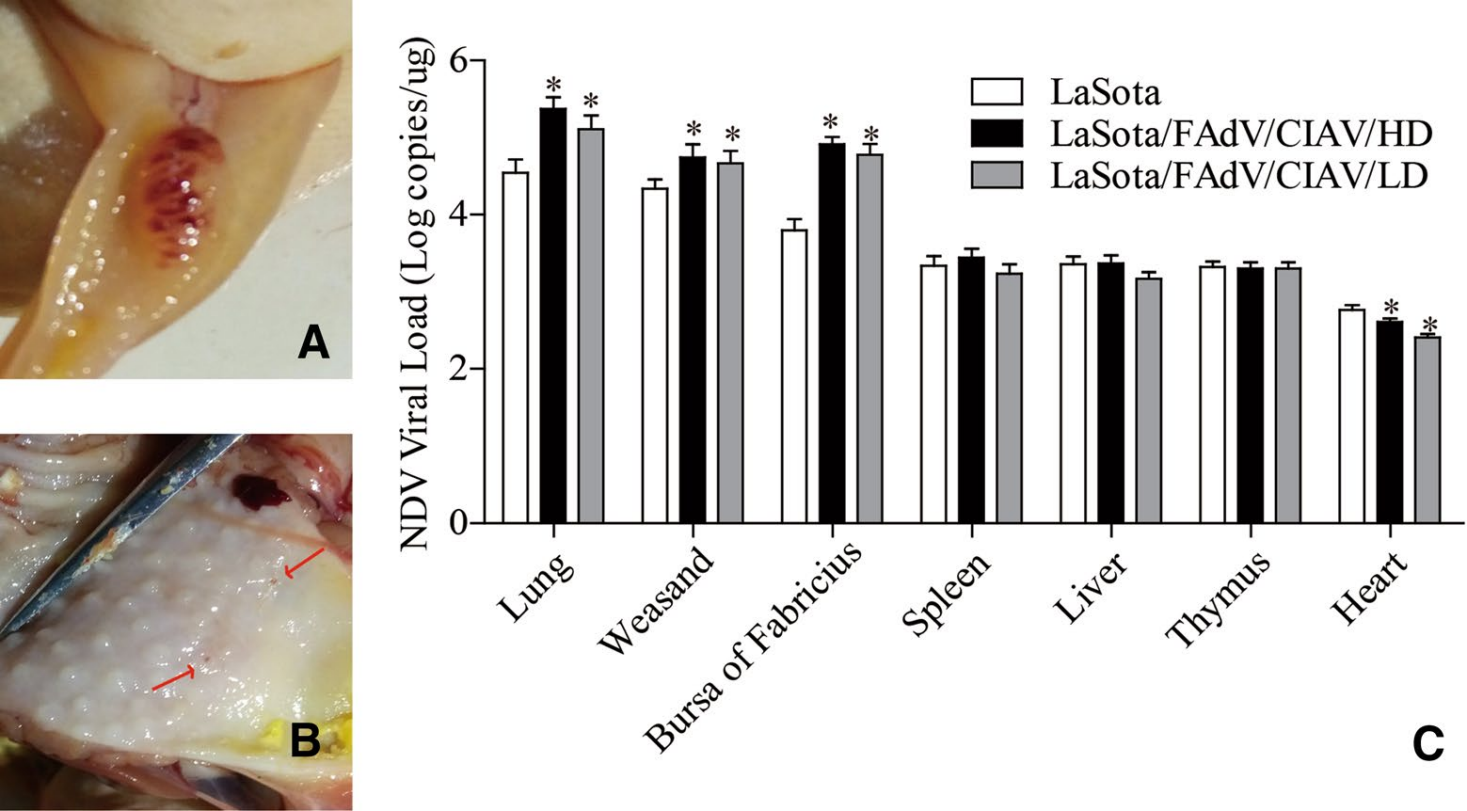
**ND indication symptoms and the distribution of LaSota in different organs**. **A** Intestinal lymphoid follicle haemorrhage and swelling; **B** glandular stomach papilla haemorrhage; **C** LaSota viral loads in different organs. Different organs were excised from three chicks randomly chosen from each group at 3 weeks of age to determine the LaSota viral load, which was represented as log_10_ copies/µL (mean value ± error). The NDV viral load value of the contaminated group was compared with that of the corresponding non-contaminated group based on Duncan‘s multiple-range test. *Indicates significant difference (*P* < 0.05) between the two experimental groups.

In the whole experiment, the NDV indication symptoms, including intestinal lymphoid follicle haemorrhage and swelling (Figure 7A) and glandular stomach papilla haemorrhage (Figure 7B), were found after using the contaminated vaccine, which demonstrated that the exogenous viruses could activate the pathogenicity of LaSota, which leads to typical ND symptoms in SPF chickens. Meanwhile, the LaSota viral loads in the lung, weasand and bursa of Fabricius of the LaSota/FAdV/CIAV/HD and LaSota/FAdV/CIAV/LD groups were significantly higher than those of the LaSota group (*P* < 0.05, Figure 7C), demonstrating that the exogenous viruses could promote the multiplication of LaSota in some organs, which might be a prerequisite for LaSota inducing ND.

### Exogenous virus contamination decreased antibody responses to vaccination against NDV

**Figure 8:**
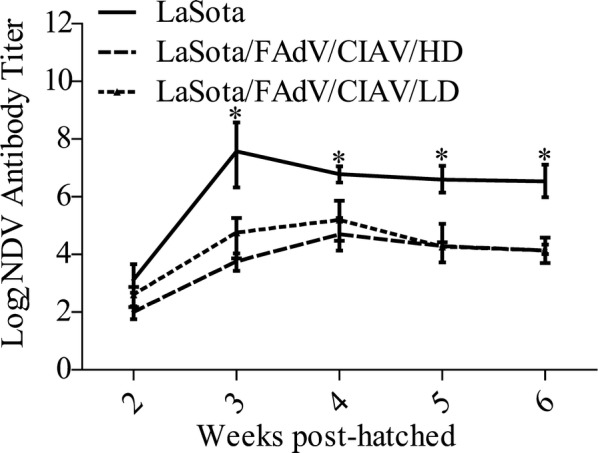
**The antibody response to vaccination with different vaccines (these data have been published in a previous study [**5**]).** The NDV antibody value of the non-contaminated group was compared with that of the corresponding contaminated group based on Duncan’s multiple-range test. *Indicates significant difference (*P* < 0.05) between the experimental groups.

Vaccine contamination is not only a source of infection for exogenous viruses but also an important interference that disturbs the production of antibodies against vaccines and significantly decreases their immunologic potency. As shown in Figure 8 (these data have been published in a previous study [5]), the NDV-antibody levels of the LaSota group were significantly higher than those of the LaSota/FAdV/CIAV/HD and LaSota/FAdV/CIAV/LD groups (*P* < 0.05). At the same time, the more contaminated the doses are, the greater the decrease of the NDV antibody titres in the early period of this experiment.

## Discussion

Exogenous virus contamination in avian-attenuated vaccine is a unique source of infection for some diseases. At present, most reports describing exogenous virus contamination mention ALV [8–10, 14], REV [7, 11, 15, 16, 19], CIAV [6, 12, 13, 17], and FAdV [18], which share the common feature of being able to vertically propagate through the chick embryo. Recently, an increased number of reports mentioned that co-infection of fowl adenovirus and chicken infectious anaemia virus was extremely common in different poultry flocks, especially in flocks with IBH–HPS [27]. However, in those farms with strict biosafety measures, most routes of virus transmission have been cut off, while the contaminated attenuated vaccines have become a vulnerable point for disease transmission. More importantly, co-contamination of FAdV and CIAV was also found in the same NDV-attenuated vaccine, which then proved to be an important reason for the outbreak of IBH–HPS on some farms [5].

Meanwhile, after being first reported in the 1930s, ND has appeared all over the world and caused huge economic losses due to its high morbidity and mortality. The World Livestock Disease Atlas surveyed 176 countries included in the OIE Animal Health Yearbooks from 2006 to 2009 and concluded that Newcastle disease is the fourth most problematic disease of poultry, behind the highly pathogenic avian influenza, avian infectious bronchitis, and low pathogenic influenza [28]. The widespread distribution of ND and the high number of annual outbreaks demonstrate that although globally used, current ND vaccines and vaccination practices alone cannot control the disease. There are many reasons for the immunization failure of NDV, and vaccine contamination might be an important factor. However, systemic assessment has rarely been performed to observe the changes of the NDV-attenuated vaccine LaSota strain with contamination and its role in exogenous virus infection.

Using FAdV and CIAV isolated from the abovementioned vaccine [5], contaminated vaccines and corresponding virus PBS diluent were artificially prepared as trial samples in this study, and simulation experiments were then performed with these samples administered through the mouth to chickens. Shockingly, IBH–HPS and consequent high mortality only appeared in the group with LaSota, FAdV and CIAV simultaneously, which demonstrated that LaSota is a crucial factor for the exogenous viruses infecting chickens and inducing severe disease after using the contaminated vaccine orally. At the same time, such synergistic reactions also aggravated the inhibitory action for BW growth of SPF chickens and even enhanced one another’s capacity for immune system destruction.

Moreover, the effects of LaSota on FAdV and CIAV were significant. Especially at the early period after vaccination, the existence of the vaccine promoted the invasion of FAdV and CIAV, potentially laying the foundation for their further multiplication. Meanwhile, we conjectured that LaSota, with high adaptability and multiplication capacity in vivo, could occupy major immune system organs and then decrease their response to FAdV and CIAV, reducing the production of antibodies against them and lessening the inhibitory action on their multiplication. All of these factors enhanced the pathogenicity of FAdV and CIAV and induced severe clinical symptoms.

On the other hand, as an exogenous contaminated virus, the effects of FAdV on LaSota were more evident, decreasing the NDV antibody titres and reducing its protective effectiveness. More importantly, typical NDV indication symptoms [1], including intestinal lymphoid follicles haemorrhage and swelling as well as glandular stomach papilla haemorrhage, were found in the LaSota/FAdV/CIAV group and not in the LaSota group, which was assertive evidence that co-infection with FAdV and CIAV activated and elevated the pathogenicity of LaSota, which induced these symptoms in chickens after vaccination. Moreover, we tested the viral load of LaSota in different organs and found that with the influence of exogenous viruses, it significantly increased in lung, weasand, and bursa of Fabricius. Although it is an attenuated vaccine, the pathogenicity of LaSota with a high viral load level could not be ignored [29].

Thus, we believe that FAdV, CIAV and LaSota play respective but related roles in vivo, increasing one another’s pathogenicity in complex interactions. FAdV-4 could damage the liver, decrease protein synthesis function, reduce plasma colloid osmotic pressure, and finally increase the exudative tendency [30]. Meanwhile, the decrease of erythrocytes caused by CIAV not only substantially lowered the oxygen transport capacity of blood, induced hypoxia and compensatory rise of angiosthenia but also could cause strong immunosuppression [31]. LaSota also caused systemic inflammation and respiratory symptoms [29]. At the same time, the immune system was unable to produce enough antibodies to resist the multiplication of these viruses under their synergistic reaction, providing opportunities for them to mass multiply and then cause more serious clinical symptoms, such as IBH–HPS.

In a word, LaSota played a key role in the exogenous virus infecting chickens and causing severe diseases after the use of contaminated vaccine, while the pathogenicity of LaSota was also significantly increased under the interference of exogenous viruses, which aggravated the diseases. All of these results demonstrated that contaminated vaccines pose a huge threat to the poultry industry, but vaccine manufacturers with huge output are numerous; thus, the existing batch release procedure and supervision system may not eliminate all problems. On the other hand, owing to the use of a very few contaminated chick embryos, only some vaccines in the same batch were contaminated with exogenous viruses; hence, it is not easy for this contamination to be found in the spot check. Thus, the vaccine producers should improve their production engineering and guarantee that there is no exogenous virus in their vaccines. The poultry farm should not be excessively dependent on vaccinations to control infectious diseases; improving biosafety conditions and strengthening the quarantine and eradication of chicks would be more effective methods for epidemic control.

This study showed the interactions and enhanced pathogenicity of FAdV, CIAV and the LaSota strain in contaminated NDV-attenuated vaccine and illustrated how the exogenous virus caused infection and induced severe disease with the help of the attenuated vaccine. Specifically, the existence of LaSota promoted the invasion of FAdV and CIAV, potentially laying the foundation for their further multiplication, and elevated their viral load in some organs and blood, as well as decreased the antibodies against them. In addition, the rapid proliferation of FAdV and CIAV in vivo could inhibit humoral immunity, elevate the LaSota viral load in some organs and decrease the NDV antibody titres. Overall, the synergistic reaction of exogenous viruses and LaSota aggravated the inhibitory action for weight growth of SPF chickens, enhanced one another’s capacity for immune system destruction, promoted their multiplication in vivo, restrained antibody production, and finally induced severe clinical symptoms, even a 75% mortality rate and typical IBH–HPS in all dead chicks. This result reminds us that the damage caused by attenuated vaccine contaminated with FAdV and CIAV even at extremely low doses is significant.

## Abbreviations

ND: Newcastle disease
NDV: Newcastle disease virus
FAdV: fowl adenovirus
CIAV: chicken infectious anaemia virus
IBH–HPS: inclusion body hepatitis–hydropericardium syndrome
qPCR: quantitative polymerase chain reactions
EID_50_: 50% embryo infectious dose
SPF: specific pathogen-free
PBS: phosphate buffered saline
BWs: body weights
